# [Corrigendum] Inhibition of acid-sensing ion channel 1a attenuates acid-induced activation of autophagy via a calcium signaling pathway in articular chondrocytes

**DOI:** 10.3892/ijmm.2026.5750

**Published:** 2026-02-02

**Authors:** Wen-Fan Gao, Ya-Yun Xu, Jin-Fang Ge, Fei-Hu Chen

Int J Mol Med 43: 1778-1788, 2019; DOI: 10.3892/ijmm.2019.4085

Following the publication of the above article, an interested reader drew to the authors' attention that the control β-actin western blots shown in Figs. 2C and 5A were strikingly similar, even though the experimental conditions reported in these figures were different.

After having re-examined the original data, the authors have realized that these western blots were inadvertently included in Fig. 2C erroneously. The revised version of Fig. 2, now incorporating the correct data for the β-actin bands in Fig. 2C, is shown below. The authors confirm that the error associated with this figure did not have a significant impact on either the results or the conclusions reported in this study, and all the authors agree with the publication of this Corrigendum. The authors are grateful to the Editor of *International Journal of Molecular Medicine* for allowing them the opportunity to publish this Corrigendum; furthermore, they apologize to the readership of the Journal for any inconvenience caused.

## Figures and Tables

**Figure 2:**
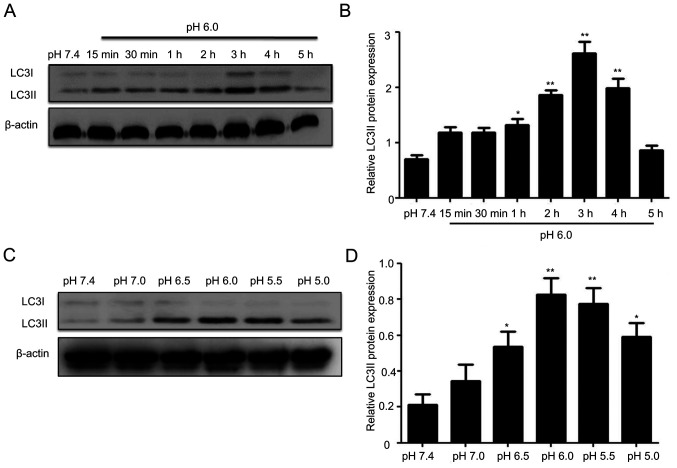
Effect of extracellular acidic solutions at different pH levels and for different time periods on the protein expression of LC3II in rat articular chondrocytes. Chondrocytes were treated without or with acid (pH 6.0) for different time periods (0-5 h) and the results (A) visualized and (B) quantified. Chondrocytes were treated without or with acid at different pH levels and the results (C) visualized and (D) quantified. The results indicated that acidic treatment had a time-and pH-dependent effect on the expression of LC3II. ^*^P<0.05 and ^**^P<0.01, vs. pH 7.4 group. LC3, microtubule-associated protein 1 light chain 3.

